# Postpartum Psychosis and Catatonia: ECT Is Still a Goal Standard Treatment

**DOI:** 10.1192/bjo.2026.11872

**Published:** 2026-06-30

**Authors:** Olubunmi Olure, Chidi Nwosu

**Affiliations:** Kent and Medway Mental Health NHS Trust, Maidstone, United Kingdom

## Abstract

**Aims:**

Pregnancy/Childbirth is a period of immense physical, emotional and social changes to women’s life due to drastic hormonal, body image changes. The immunological and circadian rhythm disturbances postpartum is compounded by potential obstetric complications.

Postpartum psychosis can arise in about 1-2/1000 live births, developing rapidly within weeks of delivery. Presentation is heterogeneous ranging from affective, psychotic and a mixture of symptoms. A smaller proportion of women can have Catatonia presenting with abnormal movements/behaviours.

**Methods:**

A 26year old woman was brought to A/E, confused and disorientated 5weeks after her first childbirth. Pregnancy was uneventful until 40weeks when developed hypertension. She was induced at 41weeks/3days after passing meconium-stained liquor. Labour lasted 3nights with postpartum haemorrhage of 1600mls. She had mastitis and UTI postpartum.

She was admitted to a mother and Baby Unit due to her deteriorating mental state. She was noted to present as mute periodically, with labile mood and abnormal movements. Other features of her presentation included thought disorder, gross confusion, delusions of paranoia and misidentification.

She experienced distressing visual, tactile and auditory hallucinations. In addition to catatonic features of echolalia, abnormal movements like walking backwards, crawling or climbing, negativism and staring with periods of immobility.

She had no previous psychiatric or medical history. She had no family history of mental illness and the rest of her psychiatric history was unremarkable with a good social support.

**Results:**

She had extensive investigations including Haematological, CSF tests, brain and body scans, EEG to rule out Autoimmune encephalitis or other organic aetiology. Results were unremarkable. She was commenced on Olanzapine with poor response despite optimisation resulting in a switch to Risperidone. She was commenced on Intra-muscular (IM) Lorazepam and responded to daily doses at 5mg though improvements were not sustained. There was a poor response to oral and sublingual Lorazepam that was tried due to concerns about frequent IM treatments.

She was subsequently treated with Electroconvulsive therapy (ECT), which led to a dramatic and rapid response. By the second session, most of the catatonic and psychotic symptoms resolved. She was subsequently discharged after 8sessions of ECT, and was able to rebuild the bond with her baby and reconnect with her family.

**Conclusion:**

Postpartum Psychosis is a psychiatric emergency with significant impairment in functioning and impact on parenting, with associated risks to self and the infant. Diagnosis and treatment must be timely. ECT remains an evidence-based treatment for rapid relief of symptoms of postpartum psychosis with Catatonia.

